# Bridging Huntington’s disease research with big data science: Harmonized neuroimaging datasets from multiple studies

**DOI:** 10.1162/imag_a_00395

**Published:** 2024-12-16

**Authors:** Dorian Pustina, Sandhitsu Das, Dan Rozelle, Hans J. Johnson, Rachael I. Scahill, Sarah J. Tabrizi, Nellie Georgiou-Karistianis, Cristina Sampaio, Andrew Wood

**Affiliations:** CHDI Management, Inc. (the company that manages the scientific activities of CHDI Foundation, Inc.), Princeton, NJ, United States; Paid Consultant, Rancho BioSciences, Rancho Santa Fe, CA, United States; Rancho BioSciences, Rancho Santa Fe, CA, United States; Department of Biomedical Engineering, University of Iowa, Iowa City, IA, United States; Department of Electrical and Computer Engineering, University of Iowa, Iowa City, IA, United States; Department of Psychiatry, University of Iowa, Iowa City, IA, United States; HD Research Centre, University College London Queen Square Institute of Neurology, UCL, London, United Kingdom; School of Psychological Sciences, Turner Institute for Brain and Mental Health, Monash University, Melbourne, VIC, Australia

**Keywords:** federation, fmri, PET, clinical, therapeutic, modeling

## Abstract

Multiple neuroimaging datasets from Huntington’s disease (HD) studies are publicly available, but these datasets are in various formats, omit imaging metadata, and sometimes contain corrupt scans. We have created a platform to curate, harmonize, and distribute neuroimaging datasets from eight different studies: TRACK-HD, TRACKOn-HD, PREDICT-HD, IMAGE-HD, HD-YAS, SHIELD-HD, PEARL-HD, and LONGPDE10. The platform is organized into three conceptual levels to serve the research community with both raw and processed data. Raw data are converted into Brain Imaging Data Structure (BIDS) format, while processed data are obtained from pipelines such as Freesurfer and fmriprep. Studies that had followed the same participants were combined. After combining studies, the final six BIDS datasets include a total of 2,216 participants and 7,073 sessions. We outline tools, principles, and recommendations for future data management in HD research.

## 1 Introduction

Huntington’s disease (HD) is a rare, genetic disorder estimated to affect nearly 400,000 people globally (Medina et al., 2022)^1^. It is caused by an expanded truplet cytosine adenine guanine (CAG) repeat in the huntingtin gene inherited from a parent (Yang et al., 2024). The disease is usually asymptomatic in childhood but typically manifests in mid adulthood with motor, cognitive, and neuropsychiatric symptoms that gradually worsen during a 10–20 year span, and ultimately leads to death. The long affliction after symptom manifestation has a devastating impact on the life of patients and their loved ones.

Although the cause of HD has been pinpointed to a single genetic mutation, HD remains an elusive problem for the biomedical community. The exact reason why specific neurons die, typically medium spiny neurons in the striatum, is not fully understood. The substantial loss of neurons before clinical manifestation suggests that therapeutics should intervene early in the disease, and neuroimaging markers currently show the best potential to help track progression and treatment efficacy in this premanifest phase (Scahill et al., 2020).

Researchers follow three invariable steps when analyzing imaging data: data organization, image processing, and statistical analyses. Although these steps are the same, each project follows a somewhat different path when combining data, choosing processing pipelines, controlling data quality, and selecting a statistical model (seeFig. 1A). The heterogeneity of research steps has been shown to produce inconsistent conclusions (Botvinik-Nezer et al., 2020). On the other hand, studies that rely on small samples are inevitably biased to find significant results only if the effect size is large, which leads to inflated, unrealistic conclusions that mislead the community (Begley & Ioannidis, 2015;Szucs & Ioannidis, 2020;Turner et al., 2018). In order to collect enough data for robust research, HD studies must run for many years across multiple sites.

**Fig. 1.**
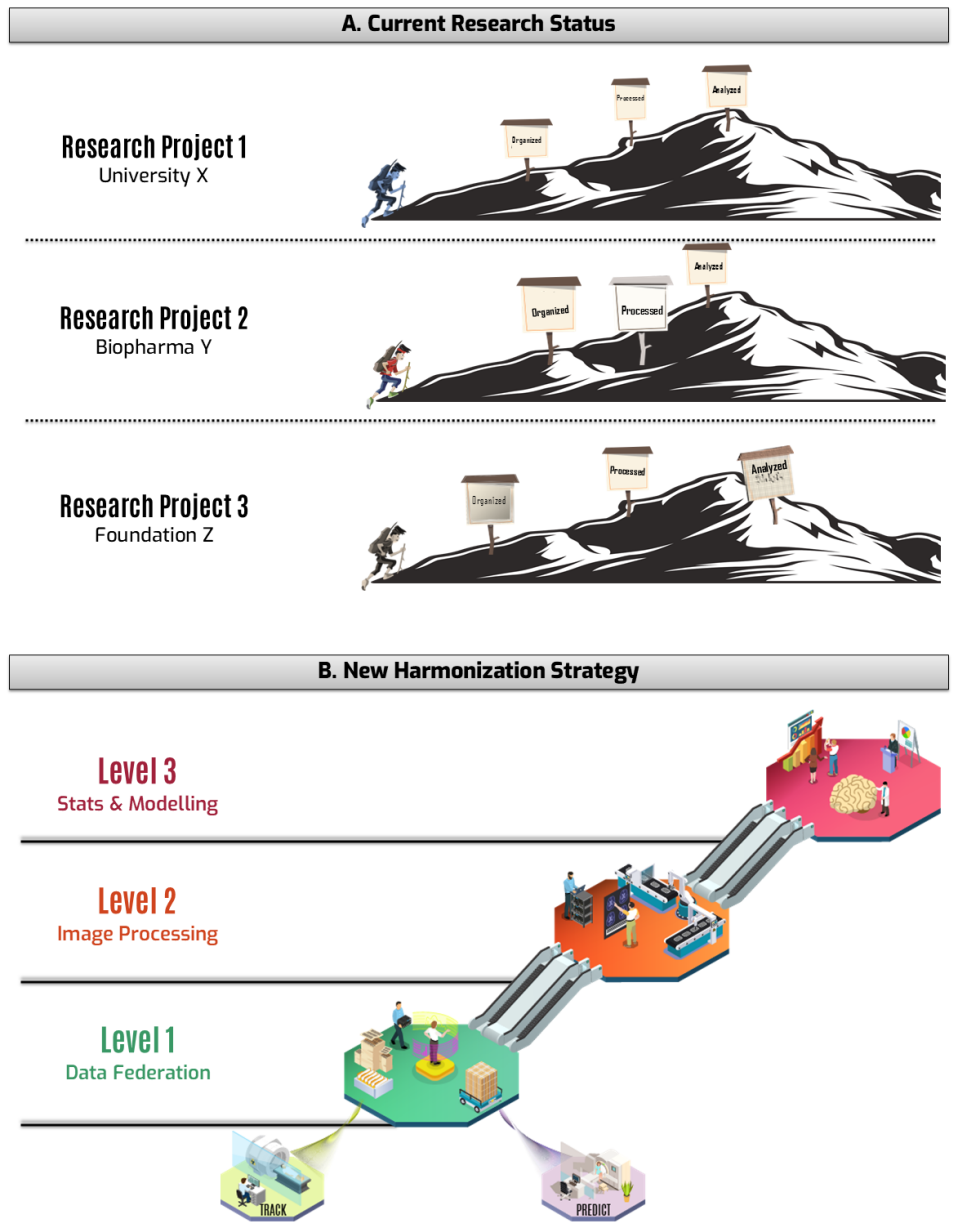
Conceptual depiction of current research status (A) and our harmonization platform (B). The three steps at the top (Organized, Processed, Analyzed) are transformed in three levels at the bottom.

Fortunately, several studies have been conducted in the past, and their data are available for public sharing (e.g., TRACK-HD, TRACKOn-HD, PREDICT-HD, IMAGE-HD, etc). Several groups have used these datasets to build staging models, investigate mechanisms of progression, and guide future clinical trials (Abeyasinghe et al., 2021;Kinnunen et al., 2021;Tabrizi et al., 2022). However, the heterogeneous formats of these datasets do not allow for effective integration to enable big-data analyses and cross-study validations. Some of them miss important metadata or include corrupt data. For example, a preliminary PREDICT-HD dataset used a defacing algorithm that compromised many scans, the IMAGE-HD data do not contain the acquisition parameters, and the TRACKOn-HD study does not include the fMRI onset times for individual participants. Even when data from various studies are combined by a research group during a research project, the combined dataset is rarely shared at the end of the project. New projects could benefit greatly from using imaging datasets that are consistently processed and contain appropriate documentation.

To address these issues, we conducted a multi-year project to curate, harmonize, and distribute imaging datasets from various HD studies. The project grew into a full harmonization platform that follows specific guidelines, tools, and outputs.

## 2 Methods

We designed three levels of data curation that mirror the research steps in a more organized fashion (Fig. 1B). Each level produces data that are ready for input at the next level, and, more importantly, this approach allows researchers to retrieve data at the level they need. Below is a conceptual description of each level.

### 2.1 Level 1 harmonization: raw data

At this level, raw data are converted, formatted, and reorganized in a common standard. Before choosing the harmonization standard, we considered the following existing solutions: the Neuroinformatics Database (NIDB,https://www.nitrc.org/projects/nidb/), Flywheel (https://flywheel.io/), XNAT (https://www.xnat.org/), DICOM-PACS (https://doi.org/10.1136%2Fadc.83.1.82), and Brain Image Data Structure (BIDS,https://bids.neuroimaging.io/). Some of these tools require a dedicated software, have restrictive licenses, need monthly payments, rely on remote data hosting, or require ongoing maintenance. The BIDS standard was determined to be the most appropriate for our needs. BIDS follows an elegant, simple approach that does not require software to hold the data. The data and metadata are simple files saved on a storage device. They are harmonized by using a specific structure of folders and filenames. For example, structural data are stored in a folder named “anat” while fMRI data are stored in folders named “bold”; filenames contain key information on the participant, session, and type of scan (e.g., “sub-T00000_ses-01_T1w.nii.gz”). Since the folders and files contain all the key information, the user can explore the dataset by simply browsing the folder structure. Scans are stored in Niftii files, while their metadata are stored in corresponding JavaScript Object Notation (JSON) files. BIDS can accept a wide range of data types, including MRI, PET, electro encephalography, and genetic data; its flexibility has made it widely adopted in the neuroimaging community, with various tools available to convert large popular datasets into BIDS (Routier et al., 2021). Importantly, BIDS datasets can be readily processed with BIDS apps, which consist of pipelines that recognize and process BIDS datasets automatically (for a complete list, seehttps://bids-apps.neuroimaging.io/apps/).

Please note that the conversion in BIDS format entailed only a transformation of the “format” of the data (e.g., file organization structure). We did not change or modify the “content” of any dataset, such as voxel values, variable names, etc., and did not apply any harmonization of scans between sites or studies.

### 2.2 Level 2 harmonization: image processing

The amount of work and resources needed to process imaging data is not trivial. Pipelines are often complex and require thorough understanding on how to troubleshoot and perform effective quality control (QC). Often, researchers are interested in testing hypotheses and building models using the final scores (e.g., volume measurements) without being distracted by the intricacies of image processing. To address this need, at the second level we prepare processed variants of the raw data. At this level, we run pipelines, QC the outputs, resolve failures, and aggregate scores in tables ready for consumption. The number of available pipelines is very large^2^, therefore we focused on running pipelines that are popular in HD research. Additional pipelines can be added in the future, while research groups can contribute in the future by providing their processed variants for each dataset. When processing imaging data, we tried to use singularity/docker containers in order to allow full harmonization of processing across datasets independently of the computing environment. Importantly, processed data in Level 2 are linked to a specific version of data in Level 1 such that we can monitor data provenance at every level.

### 2.3 Level 3 harmonization: statistical analysis

At this level, imaging scores are used to create statistical or machine-learning models. This is typically the last step of research and is done independently without needing extensive harmonization. For this reason, we do not discuss this level in detail. However, some statistical models were made available in the past (e.g., the HD-ISS calculator:https://enroll-hd.org/calc/html_basic.htm).

### 2.4 Phenotypic data integration

Phenotypic data consist of clinical, demographic, cognitive, or genetic data. These data are as critical for the analysis as the imaging data themselves. Without clinical, demographic, or cognitive data there would be no groups and no measurements to correlate with the imaging scores.

Just like imaging data, phenotypic data are heterogenous between studies. Each study may collect different demographic details, different clinical tests, or different cognitive scores. Even when tests appear to be similar, their administration may be different between studies. As a result, matching phenotypic variables among studies requires protocol reviews, expert comparison between clinical batteries, and access to original study teams who administered the test. This work was beyond the scope of our project, and we did not attempt to fully harmonize phenotypic data. However, we identified and resolved three issues that may limit future harmonization efforts. First, the tables accepted by BIDS (in the “phenotype” folder) include all the subjects. They are easy to analyze but difficult to update if the dataset changes (e.g., due to consent withdrawal by a participant). Second, these tables are not designed to hold source information on where the variable came from, which form contained it, what was the original variable name in the study, etc. Third, BIDS tables do not allow linking variables between studies (e.g., to combine “sex” and “gender”) without losing the original name.

**Fig. 2.**
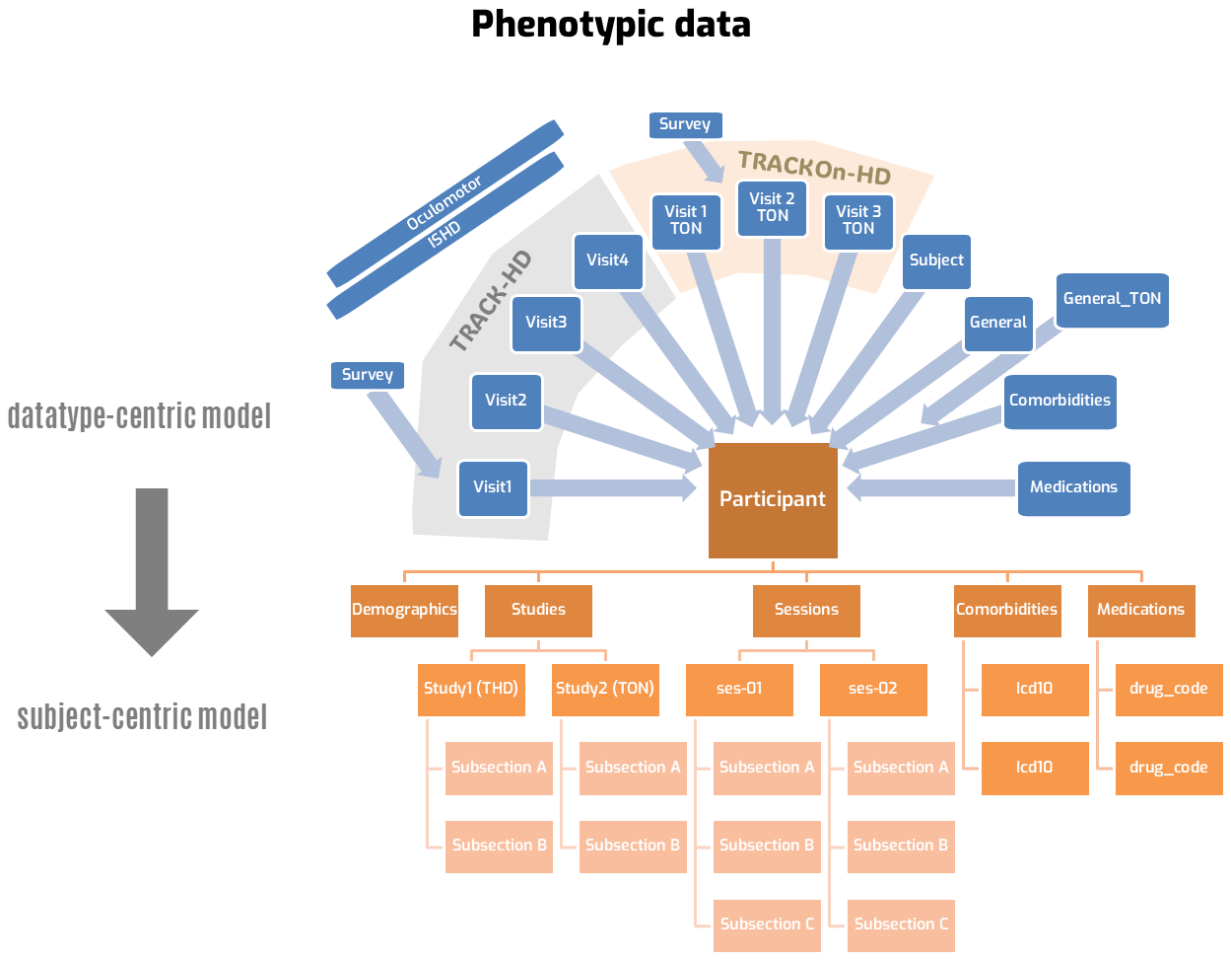
Schematic depiction of the transformation of phenotypic data from a set of tables (top) to a single-participant hierarchical JSON structure (bottom).

To resolve these issues, we designed a new approach to phenotypic data integration in BIDS. In the first step, we deconstruct the phenotypic tables to extract all the data for each individual participant. We place the phenotypic data of the participant in a subject-specific JSON file, under the participant’s imaging folder,*together*with the imaging data. The JSON file contains a hierarchical structure where major domains are on top while subsections describe more refined categories. For example, the “demographic” section contains information such as ID, age, sex, etc., which do not change; the “sessions” section includes scores collected at every study visit. The JSON file is plain text and can be read or manipulated with readily available tools.Figure 2depicts the conceptual transformation of the phenotypic data for the TRACK studies.

A single python script (phenodata.py) is used to parse the participants’ JSON files and generate tables. When calling phenodata.py, the researcher can request a subset of variables needed for a specific study, or all the variables in the dataset, which produces multiple tables. Using this approach, we automatically place the phenotypic tables in the “phenotype” BIDS folder. Since both imaging and phenotypic data are originally stored in the participant folder, updating the dataset is as simple as adding or removing a subject folder and running the phenodata.py tool again. Importantly, this approach does not substitute the BIDS standard but complements it with subject-specific files to streamline data management.

Another benefit of the new approach is the inclusion of*variable aliases*in the phenotypic data. For example, we can easily add the alias “gender” under the variable “sex”. The researcher can generate tables using any name of the variable, either original or alias. We already received and integrated variable aliases for the IMAGE-HD study. These aliases complement the original variable names collected in the study with new variable names approved by the Clinical Data Interchange Standards Consortium (CDISC) for the same data.

Lastly, the new phenotypic data storage can provide information about the provenance of the data, that is, where the data came from, what was the original variable name, what was the file that contained it, what were the original codes accepted, etc. All this information is stored within the data description file provided with the dataset.

### 2.5 Data versioning

Keeping track of data changes is critical when curating datasets. Daily progress must be saved, and the history of changes done to single files must be trackable. Mistakes happen all the time when managing data. For example, we recently discovered that even the latest PREDICT-HD dataset which has been cleaned and used for many years includes a mislabeled longitudinal scan for a participant which belongs to a different participant. We can only expect mistakes to happen and for this reason it is important to keep track of dataset versions all the time.

We use two versioning mechanisms: (1) a day-to-day versioning to track the harmonization process and (2) a final registration of datasets before public distribution. For day-to-day versioning we use Datalad, an open-source software based on the popular Git version control system (Halchenko et al., 2021). Datalad can monitor file changes, lock files from accidental modification, and move back and forth in time between saved versions. Importantly, it allows researchers to work independently on copies (or clones) of the dataset and merge the modifications later.

At the final registration for public distribution, we export a copy of the dataset from Datalad (e.g., TRACK_bids_master-v2.0) and assign a number-tag for publication reference (e.g., DATA-00000928).

### 2.6 Data deidentification/pseudonymization

A key aspect of the harmonization effort was the removal of potentially identifying information. Identifying information is any piece of data that can be used to restrict or pinpoint the search for a particular participant, such as date of birth, visit date, time of scan, geographical location, scanning facility, and scanner serial number.

To remove identifying information, we reviewed DICOM headers and JSON metadata. All dates were replaced with the number of days in the study where day 0 is the screening or baseline visit. Scan times were rounded to the nearest quarter hour. We recoded metadata that can identify the geographical location of the participant from the MRI information, such as institution name, institution address, and scanner serial number. Recoding consisted in assigning an arbitrary letter (e.g., Institution “Y”) instead of the original information (e.g., “Hopital_de_la_Pitie_Salpetriere”). We also recoded the participant IDs to create an additional barrier that prevents study sites from recovering the HD gene status of their own participants.

Facial features can potentially be reconstructed from MRI scans and help identify participants. The scientific community is becoming increasingly aware of this risk and has proposed to deface MRI data before public sharing (Eke et al., 2021). However, defacing changes the images in ways that often prevent common analysis tools from working properly (Gao et al., 2023;Rubbert et al., 2022). In some cases, the data itself is compromised and rendered unusable. For example, a previous release of the PREDICT-HD dataset used a primitive defacing algorithm which compromised many scans. The dataset was distributed for several years before an alternate defacing mechanism was used to create the current BIDS PREDICT-HD dataset described in this manuscript. Given the risks of defacing, the controlled access to data via data use agreements, and the fact that most data have been distributed before without being defaced, we did not apply defacing to previously distributed datasets.

Phenotypic data underwent separate assessment of identification risks. Dates and other potentially identifying information were removed.

## 3 Results

**Fig. 3.**
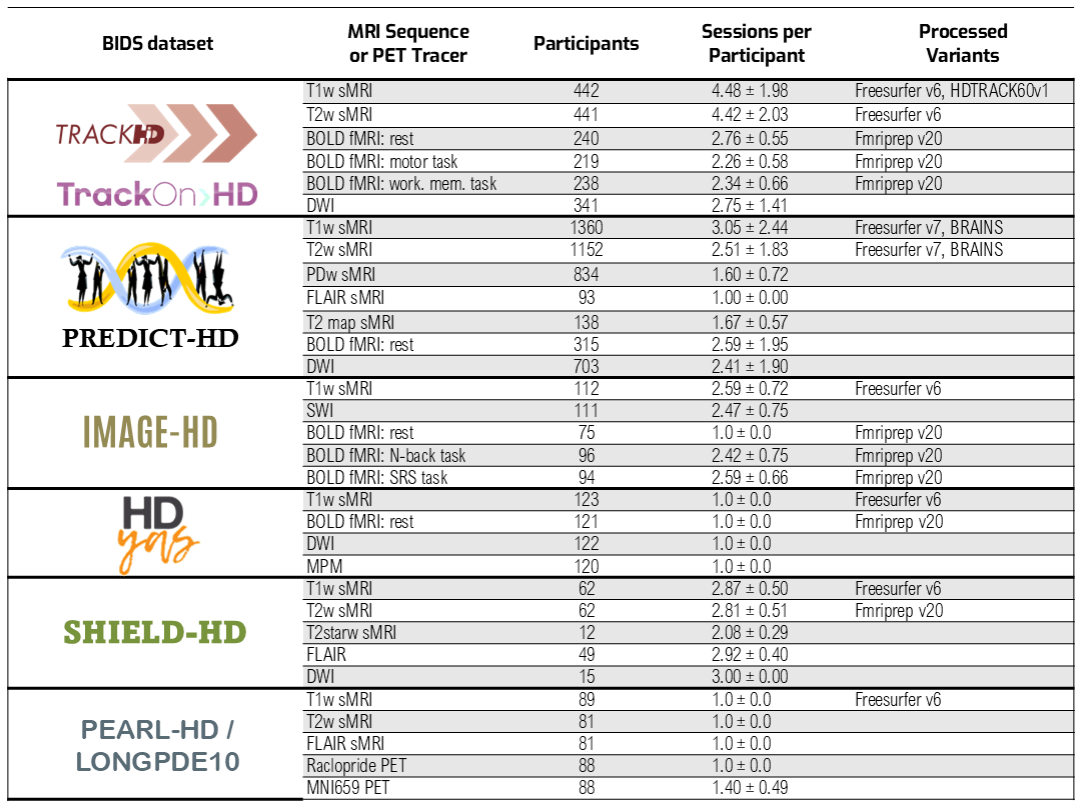
Summary of the main MRI scan types from each BIDS dataset. Failed QC scans are not counted. The rightmost column shows preprocessed variants. Acronyms: BOLD = blood oxygen level dependent; DWI = diffusion weighted imaging; FLAIR = fluid attenuated inversion recovery; fMRI = functional MRI scan type; MNI659 PET = radioactive tracer targetting Phosphodiesterase 10A enzyme; MPM = multi parametric mapping; PDw = proton density weighted; PET = positron emission tomography; Raclopride PET = radioactive tracer targetting D2/D3 dopamine receptors; sMRI = structural MRI scan type; SWI = susceptibility weighted imaging; T1w = T1 weighted; T2w = T2 weighted.

At the time of writing, we have curated data from eight HD studies; all are natural history studies with no intervention. Some of the studies were formally separate but practically longitudinal acquisitions of the same participants, so we combined these studies into single BIDS datasets. The final outcome consists of six final BIDS datasets with combined counts of 2,216 participants and 7,073 sessions. All data were collected and distributed according to the participants’ provided informed consent, and all studies underwent ethical approval at the respective Institutional Review Board of the site or country where the study was conducted.Figure 3shows the breakdown of MRI scans available for each dataset.

### 3.1 BIDS TRACK-HD/ON

This dataset contains combined data for the TRACK-HD and TRACKOn-HD studies. The TRACK-HD study recruited healthy controls, clinical symptomatic HD patients, and pre-symptomatic HD gene carriers. Data were collected at four longitudinal visits, including a baseline and three annual visits. The TRACKOn-HD study collected data for three additional years from the healthy controls and pre-symptomatic participants carried over from the TRACK-HD study. The two studies ran between 2008 and 2014 and collected imaging data at the same four sites (three sites in Europe and 1 site in North America). The institution providing the data was University College London.

CHDI received the image data in DICOM format. During the conversion process, we identified missing data and recovered some of them by opening old archives and contacting the study team. Most of the missing data was recovered successfully. For fMRI data, we contacted expert researchers familiar with the data and recovered information on stimulus onset times and response times (Kloppel et al., 2015). The DWI data had a different number of diffusion orientations between sessions and subjects; we used the “acq-“BIDS entity to expose this information (e.g., “sub-123456789_ses-07_acq-multishelldir8_run-01_dwi.nii.gz”). All the available imaging data was converted into BIDS, except Amide CEST. The latter was considered of low quality by experts familiar with the TRACKOn-HD data, and there is no CEST BIDS standard yet; the CEST data may be added later.

In general, the quality of the imaging data in the TRACK studies is very good, thanks to the rigorous study conduct, the small number of sites, and the oversight of a contract research organization (CRO). Details on imaging protocol and procedures for the TRACK studies can be found in the literature (Tabrizi et al., 2009,^2011^,^2012^; “Track-HD Study Protocol,” 2010).

During study conduct, the imaging data were manually inspected and rated by the CRO. We included these ratings as part of the BIDS sidecar JSON files in the dataset and marked the scans that failed such QC with the suffix *BAD (e.g., sub-123456789_ses-01_run-01_BADT1w.nii.gz). We did not QC the data again and usually integrated all the data in BIDS format exactly as it was received. However, we overruled the original QC for two anatomical scans marked as fail, because they were of good quality and their removal would prevent the usage of other modalities that rely on anatomical reference scans (e.g., fMRI and DWI). It is important to note that QC ratings reflect the opinion of the rater, and ratings can be overruled depending on the project needs. Researchers may want to consider QC-ing the data on their own based on their project aims. The existing *BAD scans are only a first barrier of defense to avoid their use when running automatic pipelines.

The two TRACK studies contain a rich phenotypic dataset with thousands of clinical, cognitive, and demographic scores. Variables that were the same for the two TRACK studies were harmonized and condensed into a single variable. The reorganization reduced the total number of phenotypic variables from ~5,000 to ~1,800. For each variable, we included source information of the file or form that contained it.

The final BIDS dataset contains combined data for a maximum of seven imaging sessions per participant. It includes 2024 sessions from 446 participants. Two participants have only phenotypic data and no imaging data. The remaining 444 participants include 156 healthy controls, 165 premanifest HD, and 123 HD individuals. Further details on the BIDS conversion process can be found in documentation included in the BIDS repository.

At Level 2, we processed anatomical data with Freesurfer v6 (longitudinal pipeline) (Reuter et al., 2012), which produced scores for volumes, cortical thickness, curvature, gyrification, etc. Three different builds of Freesurfer v6 were used due to processing happening in the span of 3 years. Freesurfer segmentations were manually QC-ed by the first author to identify obvious failures and verify the accuracy of caudate and putamen segmentations. The QC was conducted on the Desikan-Killiany parcellation (aparc.DKTatlas+aseg.mgz) produced by the Freesurfer cross-sectional pipeline; the regional contours and missegmentations were more clearly visible in the cross-sectional than the longitudinal parcellation. During the QC process, the rater loaded the files in ITK-SNAP software and flashed repeatedly the segmentation to detect anomalies. The rater used a 5-point rating scale to score the QC outcome (1-2 = fail, 3-5 = pass) and included an additional comment for every QC. The rating scale was created ad-hoc and was not based on any existing QC protocol. The meaning of scores is the following: 1-failed and unrecoverable (e.g., bad scan); 2-fail but recoverable (e.g., bad segmentation); 3-pass but suboptimal or borderline acceptable; 4-pass and very good quality; and 5-pass, perfect segmentation. During the review, we noticed a systematic missegmentation of the lateral border of putamen which formed a bulge from the inclusion of non-putaminal gray matter (i.e., claustrum). Since this issue was found in all the segmentations, we never gave a QC rating of 5, and the best rating given was 4. Overall, the QC process was quick (median 12 seconds, range 6–100 seconds). After QC-ing the Freesurfer segmentations, we aggregated all morphometric scores and QC values into comprehensive tables for public distribution. The raw Freesurfer data (including vertex-wise maps, thickness maps, etc,) are also available for distribution.

The HDTRACK60v1 anatomical template was created at Level 2 of the platform. The template was created by averaging T1w scans from 60 participants: 18 premanifest HD, 19 manifest HD, and 23 healthy controls. We attempted to balance the groups by site, sex, clinical status, and decade of age (Table 1). To make this template useful in processing pipelines, we computed tissue probability maps, head and brain masks, and organized the files in TemplateFlow format. To our knowledge, this is the first MRI template created from HD and healthy individuals. Its adoption may reduce potential bias stemming from the use of healthy population templates which have larger striatal regions than the HD population.

**Table 1.** Overview of participants averaged in the HDTRACK60v1 template.

Clinical status	N	Age (years)	Gender (F/M)
Control	19	48 ± 12.4	12/7
Premanifest	18	42 ± 12.5	9/9
Early HD	23	49 ± 10.8	12/11
Total	60	47 ± 12.1	33/27

We processed fMRI data with fmriprep: a comprehensive, open source, free pipeline developed at the Center for Reproducibility of Neuroimaging at the University of Stanford (Esteban et al., 2019). We had to process the data twice—first with fmriprep v1.5.9 and then with fmriprep v.20.2.7—since we discovered that v1.5.9 was affected by a bug that displaces the images in space^3^. The processed fMRI data are output in the following spaces:

MNI152NLin2009cAsym, OASIS30ANTs, HDTRACK60v1, fsaverage, fsaverage5, T1w, and native space.

### 3.2 BIDS PREDICT-HD

The PREDICT-HD study is technically the largest study to date both in terms of sample size (N>1300), number of sites (~32), and length of data collection activity (Paulsen et al., 2006,^2008^,^2010^). It collected data from clinically premanifest HD gene carriers and healthy controls. The study initiated in 2002 and recently received further funding to collect additional data (ClinicalTrials.gov ID NCT00051324). Due to the extremely long data collection efforts, the PREDICT-HD data are also the most heterogeneous as it adapted to the needs of several grant cycles, several scanner upgrades, and other inevitable changes that occur over a 20-year data collection period. We received the data from the University of Iowa.

The data from PREDICT-HD have been shared before through various channels (e.g., dbGaP,https://www.ncbi.nlm.nih.gov/projects/gap/cgi-bin/study.cgi?study_id=phs000222.v3.p2). A previous cut of this dataset contained many compromised scans due to primitive defacing applied. The new BIDS PREDICT-HD dataset was prepared at the University of Iowa in collaboration with CHDI Foundation.

Face blurring was used to deidentify participants as a mechanism to minimize the impact of post processing tools. Scans that did not pass the QC were marked with the suffix *BAD. The metadata were cleaned from sensitive information, such as site location, scanner ID, exact scan time, etc. The resulting BIDS PREDICT-HD dataset contains 1,377 participants and 4,329 sessions. These include 297 healthy controls, 1,072 HD gene carriers, and 8 participants with unkown genotype. The HD gene carriers are largely premanifest individuals grouped by low (N = 287), medium (N = 358), and high (N = 427) the CAG x age product (CAP). Note that BIDS sessions in this dataset are not equally spaced longitudinally; some sessions were collected within a short time frame from each other. The phenotypic data underwent cleanup and preparation by researchers at the University of Iowa in parallel with the imaging data. Phenotypic files were plain tables in BIDS format; we did not build subject-specific JSON files like we did in TRACK.

Level 2 processing focused on anatomical data, specifically the Freesurfer v7 and BRAINS Autoworkup pipelines (Young Kim & Johnson, 2013). Coordinates of anatomical landmarks (e.g., eyes, anterior commissure, posterior commissure) were also included in processed data (Ghayoor et al., 2018). Freesurfer v7 failures were fixed when possible and recorded in the documentation when not possible. Given the recently discovered discrepancies between Freesurfer v6 and Freesurfer v7 measurements (Filip et al., 2022;Knights et al., 2024), we also make available scores from Freesurfer v6 obtained from a previous cut of PREDICT-HD which were used to build the HD Integrated Staging System (Tabrizi et al., 2022).

In general, the data quality in the PREDICT-HD study is inconsistent across sites. The magnetic field strength varies as sites upgraded hardware; the field of view was not mandated to be oriented along the AC-PC line. Scanners changed across longitudinal visits when a site underwent hardware upgrades. We invite researchers to consider carefully the scanner upgrades during analyses since volumetric measurement bias is closely related to scanner hardware. To some degree, the above issues are related to the large number of sites and the long study duration, which inevitably lead to site hardware changes.

### 3.3 BIDS IMAGE-HD

The IMAGE-HD study collected data at a single site (Monash University). Participants underwent three MRI visits in total, with 18 months gap between visits (Dominguez et al., 2016;Georgiou-Karistianis et al., 2014;Wilkes et al., 2023). We received imaging data already converted in NIFTI format, without any imaging metadata. We also received a small number of DICOM files for reference. To create JSON files, we retrieved metadata from DICOM headers and cross-referenced them with previous publications. This approach was generally successful, except for the diffusion data. We found that the diffusion vectors provided in bval/bvec files did not match the vectors inside the DICOM files; some vectors were flipped 180°, and others were shifted in space. We attempted to identify the reason for this discrepancy without success. Our attempts included the identification of the conversion software, the retrieval of all original DICOM files from the institution, and consulting with the imaging community. Since we did not have confidence that the DWI data are accurate, we decided to exclude them in the BIDS dataset; the DWI data may be added later.

Regarding fMRI data, some acquisition metadata were recovered by contacting researchers who analyzed them in the past (Poudel et al., 2015). FMRI JSON files were completed with all the acquisition parameters necessary to run fmriprep.

The phenotypic data in this study contain limited information. We converted the phenotypic data in subject-specific JSON files and generated the BIDS-compatible phenotypic tables.

The final BIDS IMAGE-HD dataset contains data from 119 participants and 290 sessions. Seven participants had only phenotypic and no imaging data. Of the remaining 112 participants, 36 are healthy controls, 40 are premanifest HD, and 36 are manifest HD individuals. The quality of the scans in IMAGE-HD is generally good and consistent between visits.

For Level 2 processing, we ran Freesurfer v6 and fmriprep v20.2.7. The same QC and data aggregation was conducted as for BIDS TRACK-HD/ON.

### 3.4 BIDS HD-YAS

HD-YAS is a currently ongoing study conducted at University College London (single site) to collect biofluid and imaging data from young adults carrying the HD genetic mutation (Johnson et al., 2021;Scahill et al., 2020;Zeun et al., 2018). We obtained and curated the available baseline data of this study collected between 2017 and 2019.

We received the data in DICOM format and collaborated with the study team to retrieve some missing data. Most of the conversion process went smoothly; we discovered that some metadata for cutting-edge sequences (in this case, fMRI and multi-parametric mapping) may not be inside conventional DICOM headers and need special extrapolation for integration in BIDS format. Identifying and including these metadata required multiple interactions with study experts (i.e., Dr. Martina Callaghan).

The HD-YAS phenotypic data were integrated in the BIDS dataset as regular tables, without creating subject-specific phenotypic files. The current BIDS HD-YAS dataset includes 123 participants and 123 sessions. The group assignment for participants is 62 premanifest HD and 61 controls.

At Level 2, we processed structural data with Freesurfer v6 and fMRI data with fmriprep v20.2.7.

### 3.5 BIDS SHIELD-HD

The SHIELD-HD study collected imaging data from 70 participants at ten different sites for approximately 2 years (ClinicalTrials.gov ID NCT04406636). The study was sponsored by a biotech company in preparation for a future clinical trial. A commercial CRO received the data from the sites, QC-ed them, and delivered them in DICOM format.

This dataset was converted without major impediments, and the CRO was available to clarify questions and retrieve information about the study. The study protocol included the optional collection of diffusion MRI data, but this scan was not reviewed by the CRO. Unfortunately, the sites that collected diffusion used a short clinical sequence with three diffusion orientations instead of the recommended multishell sequence prescribed in the imaging manial. We included these DWI data in the BIDS dataset despite their limited research value.

Data from eight participants could not be publicly distributed due to limitations of the original consent form. The final BIDS SHIELD-HD dataset contains data from 62 HD gene expansion carriers and 183 imaging sessions; no healthy controls were recruited in this study. Participants formed three similarly sized groups (N = 19, 21, 22) at gradually more advanced levels of progression as measured by CAP scores. The phenotypic data were integrated directly in BIDS tables without creating subject-specific JSON files.

At Level 2, we processed anatomical data with Freesurfer v6, and created the aggregated Freesurfer tables.

### 3.6 BIDS PEARL-HD/LONGPDE10

The PEARL-HD study (ClinicalTrials.gov ID NCT02061722) collected structural MRI data and PET data with two radioligands: (1) [18F]MNI-659 to measure levels of Phosphodiesterase 10 A enzyme, and (2) [11 C]raclopride to measure levels of D2/D3 dopamine receptors. The study included HD gene carriers and normative controls. The LONGPDE10 study (ClinicalTrials.gov ID NCT02956148) continued follow-up PET collection of [18F]MNI-659 in HD gene carriers 1 year later; no MRI data were collected in LONGPDE10 (Fazio et al., 2020).

We received the MRI data in DICOM format, and PET data in ECAT7 format. The data from both studies were deidentified and placed in the same BIDS dataset. PET data were placed in BIDS folders in the same ECAT7 format instead of Nifti files since the PET BIDS standard was still under development at the time of conversion (Norgaard et al., 2022). The final dataset includes 89 participants and 124 sessions. The sample is composed of 45 healthy controls and 44 HD gene expansion carriers at various progression levels. Phenotypic data from these studies are still under curation and will be included in the final BIDS dataset.

## 4 Discussion

In this work, we conceptualized levels of data curation corresponding to three research steps. Raw imaging data were harmonized in BIDS datasets (Level 1), data were processed with popular pipelines (Level 2), and statistical models were enabled (Level 3). Our process followed adequate tracking of data provenance into a transparent assembly line where each expert contributes to the level they know better. We also included QC information provided by the study teams and conducted QC on thousands of Freesurfer segmentations. This is the first and largest effort to harmonize neuroimaging data in the HD field, and resulted in image data harmonization from eight studies, 2,216 participants, and 7,073 imaging sessions. The availability of these datasets comes at a time when neuroimaging research is entering the big data era when large sample sizes and cross-study validation are becoming a necessary step (Collins & Tabak, 2014;Ioannidis, 2005). More importantly, the availability of harmonized data allows researchers to ask more elaborate research questions, such as what is the profile of disease progression across multiple MRI modalities, what is the best combination of biomarkers to achieve maximal predictive power, are specific imaging biomarkers more robust than others to site and study effects, can existing segmentations be used to investigate alternative measurements like intensity-based or shape anomalies, etc? The main advantage of harmonized datasets remains the flexibility of researchers to ask new questions that are answered with lesser efforts than before.

Data sharing is as honorable objective as difficult to achieve. Despite the funding agencies mandating data sharing (e.g.,https://sharing.nih.gov/data-management-and-sharing-policy), this is not common practice yet. Curating and preparing data for public use is often an arduous task that is not part of the ladder of scientific incentives (Anger et al., 2022). In this project, we experienced first-hand the difficulties researchers have when working with unharmonized, non-consolidated datasets. The source data were frequently kept in custom folders and filenames, metadata were insufficient, sometimes scans were corrupt, and some data were lost due to insufficient documentation. Sometimes, study personnel was able to retrieve missing information, but this was not always the case; one principal investigator had moved institution and was unavailable, while in another study the researcher was available to help but had lost access to data after moving to a different institution. When sharing MRI data, the metadata inside DICOM files are highly valuable to enable correct processing, but these files are not always made available. One institution had archived DICOM data in magnetic tapes, and the retrieval was complex and ultimately not possible. One study relied on a commercial CRO to manage, curate, and store the data, but even the CRO delivered incomplete and duplicate scans that required several iterations of data transfer.

The typical reason for inadequate data management in medical research is the lack of incentives. Scientists claim that collecting, cleaning, and organizing data take over 50% of the time in research projects and are the least desirable tasks (CrowdFlower, 2017). While organizing and cleaning data for personal projects is not an exciting job, organizing data for the benefit of the community is even less interesting given that the work is not rewarded in academic circles. To resolve the issue, the scientific community will need to place more value in data management by recognizing, citing, and funding data curation work in order to enable scientists to show their skills within the scientific ecosystem itself (Pierce et al., 2019).

A key tool to improve data management in future studies is the data management plan (DMP). DMPs include information on how data will flow, where it will be stored, how it will be quality-controlled, and how it will be formatted for public sharing; DMPs are also required by funding agencies. Besides the DMP itself, it is very important to have qualified personnel that have both scientific knowledge—to be able to understand the data—and data management knowledge—to be able to make choices on data-sharing practices (Hajduk et al., 2019;Wilkinson et al., 2016). The gap between optimal and practical data management can be narrowed by a good DMP combined with good expertise/training of the personnel. If the data are properly curated during study conduct, there is less need to curate data after the study has concluded.

Another issue we encountered in this project is that modifying datasets and fixing errors is a slow process due to bureaucratic steps emerging from inter-institutional collaborations. Almost all datasets we handled had some kind of issue that needed iterations to fix, and sometimes the institution that owned the data had to intervene. It takes years sometimes for institutions to fix the errors on the data they distribute. While some of these issues can be avoided in the future with better QC of the distributed data, it is nearly impossible for any institution to produce perfect datasets that are immutable in time. For this reason, data sharing will need to account for multiple iterations of data correction until errors are addressed. Therefore, we believe that labs and institutions that share the data would benefit from mechanisms of feedback after the data are made public, particularly after the initial release.

At Level 2 of our platform, we prepared several processed variants of the datasets with popular pipelines (e.g., Freesurfer, fmriprep). It is important to stress that the choices of the pipelines we adopted do not entail an endorsement or consideration of a pipeline as the best, most optimal, or most accurate for HD research. On the contrary, we noticed systematic inaccuracies in the Freesurfer segmentations of subcortical structures, putamen in particular, which may render the pipeline problematic when researchers need precise measurement of the structure (e.g., for surgical injections). The adoption of the pipelines in our project was made simply to help the community avoid reprocessing of the data over and over again. We expect Level 2 data to be expanded in the future with other processed variants, which will allow more rigorous comparisons of pipelines with each other.

Of the eight studies we worked on, four studies were conducted at multiple sites: TRACK-HD, TRACKOn-HD, PREDICT-HD, and SHIELD-HD. With the exception of the TRACK-HD study which performed sequence optimization prior to the start (described in supplementary material inTabrizi et al. (2009)), the other studies did not perform image contrast harmonization between sites. For PREDICT-HD and SHIELD-HD, in particular, it is clearly visible that sites used different scan parameters which led to different T1-weighted contrasts. We only reformatted the existing raw data and did not intervene to modify the data in any way. The site effects will need to be removed by researchers who analyze the data.

Our current work has some known limitations. First, we did not harmonize phenotypic data, and this may limit the utility of having harmonized imaging datasets. Nonetheless, the time saved from massive processing of imaging data with BIDS apps can be dedicated to matching phenotypic data between studies. Second, the studies described here have other types of data not included in BIDS datasets, for example, genomic data. There is already an extension proposal to include genomic data in BIDS, which may help future efforts of multimodal data integration within a harmonized framework (Moreau et al., 2020).

In conclusion, we made the first leap toward bridging HD research and big data science by providing multiple curated neuroimaging datasets. Similar work has been done in other neurodegenerative diseases (De Stefano et al., 2022;Prado et al., 2023) which speaks of the importance of data federation for accelerating the pace of medical research. We will continue to expand the pool of datasets with other studies that have become available for public distribution (e.g., HD-CSF and JoHD).

## Data and Code Availability

The data described here can be obtained from CHDI Foundation (seehttps://www.enroll-hd.org/for-researchers/datasets/). The process requires signing data use agreements that prohibit participant reidentification, and verification of the computing systems that will hold the data (as per GDPR regulations,https://gdpr-info.eu/art-32-gdpr/). These steps may require time depending on the involvement of legal and information technology departments of the receiving institution. The code used to organize the data, QC the results, and create phenotypic tables is provided together with each dataset.

## Author Contributions

D.P.: designed and oversaw the project, guided the processing of all datasets, reviewed image parcellations, and wrote the manuscript; S.D.: curated and processed the imaging data from various datasets, revised the manuscript; D.R.: curated and processed the phenotypic data from various datasets, revised the manuscript; H.J.J.: curated and processed the BIDS PREDICT-HD dataset; R.I.S.: guided the curation of the BIDS TRACK-HD/ON dataset; S.J.T.: guided the curation of the BIDS TRACK-HD/ON dataset, revised the manuscript; N.G.-K.: guided the curation of the BIDS IMAGE-HD dataset; C.S.: oversaw the project, revised the manuscript; and A.W.: oversaw the project, revised the manuscript.

## Declaration of Competing Interest

The authors declare no competing interests from the results and outcome produced by this work.

## Footnotes

1 Calculated from a prevalence of 4.88/100,000 and current world population of 8.1 billion.

2 For morphometric analysis we could run Freesurfer, SPM, ANTs, BRAINS Autoworkup, MALP-EM, STEPS, FIRST, MRIStudio, Brain Shift Integral, etc.; for diffusion data we could run MRtrix, MRIstudio, ExploreDTI, DSIStudio, QSIprep, etc.

3 Seehttps://github.com/nipreps/fmriprep/issues/2307

## References

[b1] Abeyasinghe , P. M. , Long , J. D. , Razi , A. , Pustina , D. , Paulsen , J. S. , Tabrizi , S. J. , Poudel , G. R. , & Georgiou-Karistianis , N. ( 2021 ). Tracking Huntington’s disease progression using motor, functional, cognitive, and imaging markers . Mov Disord , 36 ( 10 ), 2282 – 2292 . 10.1002/mds.28650 34014005 PMC8590922

[b2] Anger , M. , Wendelborn , C. , Winkler , E. C. , & Schickhardt , C. ( 2022 ). Neither carrots nor sticks? Challenges surrounding data sharing from the perspective of research funding agencies-A qualitative expert interview study . PLoS One , 17 ( 9 ), e0273259 . 10.1371/journal.pone.0273259 36070283 PMC9451069

[b3] Begley , C. G. , & Ioannidis , J. P. ( 2015 ). Reproducibility in science: Improving the standard for basic and preclinical research . Circ Res , 116 , 116 – 126 . 10.1161/CIRCRESAHA.114.303819 25552691

[b4] Botvinik-Nezer , R. , Holzmeister , F. , Camerer , C. F. , Dreber , A. , Huber , J. , Johannesson , M. , Kirchler , M. , Iwanir , R. , Mumford , J. A. , Adcock , R. A. , Avesani , P. , Baczkowski , B. M. , Bajracharya , A. , Bakst , L. , Ball , S. , Barilari , M. , Bault , N. , Beaton , D. , Beitner , J. ,… Schonberg , T. ( 2020 ). Variability in the analysis of a single neuroimaging dataset by many teams . Nature , 582 ( 7810 ), 84 – 88 . 10.1038/s41586-020-2314-9 32483374 PMC7771346

[b5] Collins , F. S. , & Tabak , L. A. ( 2014 ). Policy: NIH plans to enhance reproducibility . Nature , 505 , 612 – 613 . http://www.ncbi.nlm.nih.gov/pubmed/24482835 24482835 10.1038/505612a PMC4058759

[b6] CrowdFlower . ( 2017 ). 2017 data scientist report . San Francisco . https://www.scribd.com/document/374992111/CrowdFlower-DataScienceReport-pdf

[b7] De Stefano , N. , Battaglini , M. , Pareto , D. , Cortese , R. , Zhang , J. , Oesingmann , N. , Prados , F. , Rocca , M. A. , Valsasina , P. , Vrenken , H. , Gandini Wheeler-Kingshott, C. A. M. , Filippi , M. , Barkhof , F. , & Rovira , À. ; MAGNIMS Study Group . ( 2022 ). MAGNIMS recommendations for harmonization of MRI data in MS multicenter studies . NeuroImage Clin , 34 , 102972 . 10.1016/j.nicl.2022.102972 35245791 PMC8892169

[b8] Dominguez , J. F. , Stout , J. C. , Poudel , G. , Churchyard , A. , Chua , P. , Egan , G. F. , & Georgiou-Karistianis , N. ( 2016 ). Multimodal imaging biomarkers in premanifest and early Huntington’s disease: 30-month IMAGE-HD data . Br J Psychiatry , 208 ( 6 ), 571 – 578 . 10.1192/bjp.bp.114.156588 26678864

[b9] Eke , D. , Aasebø , I. E. J. , Akintoye , S. , Knight , W. , Karakasidis , A. , Mikulan , E. , Ochang , P. , Ogoh , G. , Oostenveld , R. , Pigorini , A. , Stahl , B. C. , White , T. , & Zehl , L. ( 2021 ). Pseudonymisation of neuroimages and data protection: Increasing access to data while retaining scientific utility . Neuroimage Reports , 1 ( 4 ), 100053 . 10.1016/j.ynirp.2021.100053 40568426 PMC12172758

[b10] Esteban , O. , Markiewicz , C. J. , Blair , R. W. , Moodie , C. A. , Isik , A. I. , Erramuzpe , A. , Kent , J. D. , Goncalves , M. , DuPre , E. , Snyder , M. , Oya , H. , Ghosh , S. S. , Wright , J. , Durnez , J. , Poldrack , R. A. , & Gorgolewski , K. J. ( 2019 ). fMRIPrep: A robust preprocessing pipeline for functional MRI . Nat Methods , 16 ( 1 ), 111 – 116 . 10.1038/s41592-018-0235-4 30532080 PMC6319393

[b11] Fazio , P. , Fitzer-Attas , C. J. , Mrzljak , L. , Bronzova , J. , Nag , S. , Warner , J. H. , Landwehrmeyer , B. , Al-Tawil , N. , Halldin , C. , Forsberg , A. , Ware , J. , Dilda , V. , Wood , A. , Sampaio , C. , & Varrone , A. ; PEARL-HD and LONGPDE10 Study Collaborators . ( 2020 ). PET molecular imaging of phosphodiesterase 10a: An early biomarker of Huntington’s disease progression . Mov Disord , 35 ( 4 ), 606 – 615 . 10.1002/mds.27963 31967355

[b12] Filip , P. , Bednarik , P. , Eberly , L. E. , Moheet , A. , Svatkova , A. , Grohn , H. , Kumar , A. F. , Seaquist , E. R. , & Mangia , S. ( 2022 ). Different FreeSurfer versions might generate different statistical outcomes in case-control comparison studies . Neuroradiology , 64 ( 4 ), 765 – 773 . 10.1007/s00234-021-02862-0 34988592 PMC8916973

[b13] Gao , C. , Landman , B. A. , Prince , J. L. , & Carass , A. ( 2023 ). A reproducibility evaluation of the effects of MRI defacing on brain segmentation . medRxiv . 10.1101/2023.05.15.23289995 PMC10704191 38074632

[b14] Georgiou-Karistianis , N. , Stout , J. C. , Domínguez , D. J. , Carron , S. P. , Ando , A. , Churchyard , A. , Chua , P. , Bohanna , I. , Dymowski , A. R. , Poudel , G. , & Egan , G. F. ( 2014 ). Functional magnetic resonance imaging of working memory in Huntington’s disease: Cross-sectional data from the IMAGE-HD study . Hum Brain Mapp , 35 ( 5 ), 1847 – 1864 . 10.1002/hbm.22296 23913754 PMC6869353

[b15] Ghayoor , A. , Vaidya , J. G. , & Johnson , H. J. ( 2018 ). Robust automated constellation-based landmark detection in human brain imaging . Neuroimage , 170 , 471 – 481 . 10.1016/j.neuroimage.2017.04.012 28392490 PMC5630513

[b16] Hajduk , G. K. , Jamieson , N. E. , Baker , B. L. , Olesen , O. F. , & Lang , T. ( 2019 ). It is not enough that we require data to be shared; we have to make sharing easy, feasible and accessible too! BMJ Global Health , 4 ( 4 ), e001550 . 10.1136/bmjgh-2019-001550 PMC6666804 31406588

[b17] Halchenko , Y. , Meyer , K. , Poldrack , B. , Solanky , D. , Wagner , A. , Gors , J. , MacFarlane , D. , Pustina , D. , Sochat , V. , Ghosh , S. S. , Mönch , C. , Markiewicz , C. J. , Waite , L. , Shlyakhter , I. , de la Vega , A., Hayashi , S. , Häusler , C. O. , Poline , J. B. , Kadelka , T. ,… Hanke , M. ( 2021 ). DataLad: Distributed system for joint management of code, data, and their relationship . J Open Source Softw , 6 ( 63 ), 3262 . 10.21105/joss.03262 39469147 PMC11514317

[b18] Ioannidis , J. P. ( 2005 ). Why most published research findings are false . PLoS Med , 2 ( 8 ), e124 . 10.1371/journal.pmed.0020124 16060722 PMC1182327

[b19] Johnson , E. B. , Parker , C. S. , Scahill , R. I. , Gregory , S. , Papoutsi , M. , Zeun , P. , Osborne-Crowley , K. , Lowe , J. , Nair , A. , Estevez-Fraga , C. , Fayer , K. , Rees , G. , Zhang , H. , & Tabrizi , S. J. ; HD-YAS Investigators . ( 2021 ). Altered iron and myelin in premanifest Huntington’s disease more than 20 years before clinical onset: Evidence from the cross-sectional HD Young Adult Study . EBioMedicine , 65 , 103266 . 10.1016/j.ebiom.2021.103266 33706250 PMC7960938

[b20] Kinnunen , K. M. , Schwarz , A. J. , Turner , E. C. , Pustina , D. , Gantman , E. C. , Gordon , M. F. , Joules , R. , Mullin , A. P. , Scahill , R. I. , & Georgiou-Karistianis , N. ; Huntington’s Disease Regulatory Science Consortium (HD-RSC) . ( 2021 ). Volumetric MRI-based biomarkers in Huntington’s disease: An evidentiary review . Front Neurol , 12 , 712555 . 10.3389/fneur.2021.712555 34621236 PMC8490802

[b21] Kloppel , S. , Gregory , S. , Scheller , E. , Minkova , L. , Razi , A. , Durr , A. , Roos , R. A. , Leavitt , B. R. , Papoutsi , M. , Landwehrmeyer , G. B. , Reilmann , R. , Borowsky , B. , Johnson , H. , Mills , J. A. , Owen , G. , Stout , J. , Scahill , R. I. , Long , J. D. , Rees , G. , & Tabrizi , S. J. ; Track-On Investigators . ( 2015 ). Compensation in preclinical Huntington’s disease: Evidence from the track-on HD study . EBioMedicine , 2 ( 10 ), 1420 – 1429 . 10.1016/j.ebiom.2015.08.002 26629536 PMC4634199

[b22] Knights , H. , Coleman , A. , Hobbs , N. Z. , Tabrizi , S. J. , & Scahill , R. I. ( 2024 ). Freesurfer software update significantly impacts striatal volumes in the Huntington’s disease young adult study and will influence HD-ISS staging . J Huntingtons Dis , 13 ( 1 ), 77 – 90 . 10.3233/jhd-231512 38489194

[b23] Medina , A. , Mahjoub , Y. , Shaver , L. , & Pringsheim , T. ( 2022 ). Prevalence and incidence of Huntington’s disease: An updated systematic review and meta-analysis . Mov Disord , 37 ( 12 ), 2327 – 2335 . 10.1002/mds.29228 36161673 PMC10086981

[b24] Moreau , C. A. , Jean-Louis , M. , Blair , R. , Markiewicz , C. J. , Turner , J. A. , Calhoun , V. D. , Nichols , T. E. , & Pernet , C. R. ( 2020 ). The genetics-BIDS extension: Easing the search for genetic data associated with human brain imaging . Gigascience , 9 ( 10 ), giaa104 . 10.1093/gigascience/giaa104 33068112 PMC7568436

[b25] Norgaard , M. , Matheson , G. J. , Hansen , H. D. , Thomas , A. , Searle , G. , Rizzo , G. , Veronese , M. , Giacomel , A. , Yaqub , M. , Tonietto , M. , Funck , T. , Gillman , A. , Boniface , H. , Routier , A. , Dalenberg , J. R. , Betthauser , T. , Feingold , F. , Markiewicz , C. J. , Gorgolewski , K. J. ,… Ganz , M. ( 2022 ). PET-BIDS, an extension to the brain imaging data structure for positron emission tomography . Sci Data , 9 ( 1 ), 65 . 10.1038/s41597-022-01164-1 35236846 PMC8891322

[b26] Paulsen , J. S. , Hayden , M. , Stout , J. C. , Langbehn , D. R. , Aylward , E. , Ross , C. A. , Guttman , M. , Nance , M. , Kieburtz , K. , Oakes , D. , Shoulson , I. , Kayson , E. , Johnson , S. , & Penziner , E. ; Predict-HD Investigators of the Huntington Study Group . ( 2006 ). Preparing for preventive clinical trials: The Predict-HD study . Arch Neurol , 63 ( 6 ), 883 – 890 . 10.1001/archneur.63.6.883 16769871

[b27] Paulsen , J. S. , Langbehn , D. R. , Stout , J. C. , Aylward , E. , Ross , C. A. , Nance , M. , Guttman , M. , Johnson , S. , MacDonald , M. , Beglinger , L. J. , Duff , K. , Kayson , E. , Biglan , K. , Shoulson , I. , Oakes , D. , & Hayden , M. ; Predict-HD Investigators and Coordinators of the Huntington Study Group . ( 2008 ). Detection of Huntington’s disease decades before diagnosis: The Predict-HD study . J Neurol Neurosurg Psychiatry , 79 ( 8 ), 874 – 880 . 10.1136/jnnp.2007.128728 18096682 PMC2569211

[b28] Paulsen , J. S. , Nopoulos , P. C. , Aylward , E. , Ross , C. A. , Johnson , H. , Magnotta , V. A. , Juhl , A. , Pierson , R. K. , Mills , J. , Langbehn , D. , & Nance , M. ; PREDICT-HD Investigators and Coordinators of the Huntington’s Study Group (HSG) . ( 2010 ). Striatal and white matter predictors of estimated diagnosis for Huntington disease . Brain Res Bull , 82 ( 3–4 ), 201 – 207 . 10.1016/j.brainresbull.2010.04.003 20385209 PMC2892238

[b29] Pierce , H. H. , Dev , A. , Statham , E. , & Bierer , B. E. ( 2019 ). Credit data generators for data reuse . Nature , 570 ( 7759 ), 30 – 32 . 10.1038/d41586-019-01715-4 31164773

[b30] Poudel , G. R. , Driscoll , S. , Domínguez , D. J. , Stout , J. C. , Churchyard , A. , Chua , P. , Egan , G. F. , & Georgiou-Karistianis , N. ( 2015 ). Functional brain correlates of neuropsychiatric symptoms in presymptomatic Huntington’s disease: The IMAGE-HD study . J Huntingtons Dis , 4 ( 4 ), 325 – 332 . 10.3233/jhd-150154 26756589

[b31] Prado , P. , Medel , V. , Gonzalez-Gomez , R. , Sainz-Ballesteros , A. , Vidal , V. , Santamaría-García , H. , Moguilner , S. , Mejia , J. , Slachevsky , A. , Behrens , M. I. , Aguillon , D. , Lopera , F. , Parra , M. A. , Matallana , D. , Maito , M. A. , Garcia , A. M. , Custodio , N. , Funes , A. Á. , Piña-Escudero , S. ,… Ibañez , A. ( 2023 ). The BrainLat project, a multimodal neuroimaging dataset of neurodegeneration from underrepresented backgrounds . Sci Data , 10 ( 1 ), 889 . 10.1038/s41597-023-02806-8 38071313 PMC10710425

[b32] Reuter , M. , Schmansky , N. J. , Rosas , H. D. , & Fischl , B. ( 2012 ). Within-subject template estimation for unbiased longitudinal image analysis . Neuroimage , 61 ( 4 ), 1402 – 1418 . 10.1016/j.neuroimage.2012.02.084 22430496 PMC3389460

[b33] Routier , A. , Burgos , N. , Díaz , M. , Bacci , M. , Bottani , S. , El-Rifai , O. , Fontanella , S. , Gori , P. , Guillon , J. , Guyot , A. , Hassanaly , R. , Jacquemont , T. , Lu , P. , Marcoux , A. , Moreau , T. , Samper-González , J. , Teichmann , M. , Thibeau-Sutre , E. , Vaillant , G. ,… Colliot , O. ( 2021 ). Clinica: An open-source software platform for reproducible clinical neuroscience studies . Front Neuroinform , 15 , 689675 . 10.3389/fninf.2021.689675 34483871 PMC8415107

[b34] Rubbert , C. , Wolf , L. , Turowski , B. , Hedderich , D. M. , Gaser , C. , Dahnke , R. , & Caspers , J. ( 2022 ). Impact of defacing on automated brain atrophy estimation . Insights Imaging , 13 ( 1 ), 54 . 10.1186/s13244-022-01195-7 35348936 PMC8964867

[b35] Scahill , R. I. , Zeun , P. , Osborne-Crowley , K. , Johnson , E. B. , Gregory , S. , Parker , C. , Lowe , J. , Nair , A. , O’Callaghan , C. , Langley , C. , Papoutsi , M. , McColgan , P. , Estevez-Fraga , C. , Fayer , K. , Wellington , H. , Rodrigues , F. B. , Byrne , L. M. , Heselgrave , A. , Hyare , H. ,… Tabrizi , S. J. ( 2020 ). Biological and clinical characteristics of gene carriers far from predicted onset in the Huntington’s disease Young Adult Study (HD-YAS): A cross-sectional analysis . Lancet Neurol , 19 ( 6 ), 502 – 512 . 10.1016/s1474-4422(20)30143-5 32470422 PMC7254065

[b36] Szucs , D. , & Ioannidis , J. P. ( 2020 ). Sample size evolution in neuroimaging research: An evaluation of highly-cited studies (1990–2012) and of latest practices (2017-2018) in high-impact journals . Neuroimage , 221 , 117164 . 10.1016/j.neuroimage.2020.117164 32679253

[b37] Tabrizi , S. J. , Langbehn , D. R. , Leavitt , B. R. , Roos , R. A. , Durr , A. , Craufurd , D. , Kennard , C. , Hicks , S. L. , Fox , N. C. , Scahill , R. I. , Borowsky , B. , Tobin , A. J. , Rosas , H. D. , Johnson , H. , Reilmann , R. , Landwehrmeyer , B. , & Stout , J. C. ; TRACK-HD Investigators . ( 2009 ). Biological and clinical manifestations of Huntington’s disease in the longitudinal TRACK-HD study: Cross-sectional analysis of baseline data . Lancet Neurol , 8 ( 9 ), 791 – 801 . 10.1016/s1474-4422(09)70170-x 19646924 PMC3725974

[b38] Tabrizi , S. J. , Reilmann , R. , Roos , R. A. , Durr , A. , Leavitt , B. , Owen , G. , Jones , R. , Johnson , H. , Craufurd , D. , Hicks , S. L. , Kennard , C. , Landwehrmeyer , B. , Stout , J. C. , Borowsky , B. , Scahill , R. I. , Frost , C. , & Langbehn , D. R. ; TRACK-HD Investigators . ( 2012 ). Potential endpoints for clinical trials in premanifest and early Huntington’s disease in the TRACK-HD study: Analysis of 24 month observational data . Lancet Neurol , 11 ( 1 ), 42 – 53 . 10.1016/S1474-4422(11)70263-0 22137354

[b39] Tabrizi , S. J. , Scahill , R. I. , Durr , A. , Roos , R. A. , Leavitt , B. R. , Jones , R. , Landwehrmeyer , G. B. , Fox , N. C. , Johnson , H. , Hicks , S. L. , Kennard , C. , Craufurd , D. , Frost , C. , Langbehn , D. R. , Reilmann , R. , & Stout , J. C. ; TRACK-HD Investigators . ( 2011 ). Biological and clinical changes in premanifest and early stage Huntington’s disease in the TRACK-HD study: The 12-month longitudinal analysis . Lancet Neurol , 10 ( 1 ), 31 – 42 . 10.1016/s1474-4422(10)70276-3 21130037

[b40] Tabrizi , S. J. , Schobel , S. , Gantman , E. C. , Mansbach , A. , Borowsky , B. , Konstantinova , P. , Mestre , T. A. , Panagoulias , J. , Ross , C. A. , Zauderer , M. , Mullin , A. P. , Romero , K. , Sivakumaran , S. , Turner , E. C. , Long , J. D. , & Sampaio , C. ; Huntington’s Disease Regulatory Science Consortium (HD-RSC) . ( 2022 ). A biological classification of Huntington’s disease: The integrated staging system . Lancet Neurol , 21 ( 7 ), 632 – 644 . 10.1016/s1474-4422(22)00120-x 35716693

[b41] Track-HD Study Protocol v4.0 . https://www.ucl.ac.uk/ion/sites/ion/files/trackhd-study-protocol-visit_4_version_4.0.pdf . ( 2010 ). Retrieved from http://hdresearch.ucl.ac.uk/wp-content/uploads/trackhd-study-protocol-visit-1-version-1.3.pdf

[b42] Turner , B. O. , Paul , E. J. , Miller , M. B. , & Barbey , A. K. ( 2018 ). Small sample sizes reduce the replicability of task-based fMRI studies . Commun Biol , 1 ( 1 ), 62 . 10.1038/s42003-018-0073-z 30271944 PMC6123695

[b43] Wilkes , F. A. , Jakabek , D. , Walterfang , M. , Velakoulis , D. , Poudel , G. R. , Stout , J. C. , Chua , P. , Egan , G. F. , Looi , J. C. L. , & Georgiou-Karistianis , N. ( 2023 ). The shape of things to come. Mapping spatiotemporal progression of striatal morphology in Huntington disease: The IMAGE-HD study . Psychiatry Res Neuroimaging , 335 , 111717 . 10.1016/j.pscychresns.2023.111717 37751638

[b44] Wilkinson , M. D. , Dumontier , M. , Aalbersberg , I. J. , Appleton , G. , Axton , M. , Baak , A. , Blomberg , N. , Boiten , J. W. , da Silva Santos , L. B. , Bourne , P. E. , Bouwman , J. , Brookes , A. J. , Clark , T. , Crosas , M. , Dillo , I. , Dumon , O. , Edmunds , S. , Evelo , C. T. , Finkers , R. ,… Mons , B. ( 2016 ). The FAIR guiding principles for scientific data management and stewardship . Sci Data , 3 , 160018 . 10.1038/sdata.2016.18 26978244 PMC4792175

[b45] Yang , X. W. , Heiman , M. , & Thompson , L. M. ( 2024 ). Huntington’s disease: Pathogenic mechanisms and implications for therapeutics . Elsevier Science . 10.1016/C2021-0-03525-2

[b46] Young Kim, E. , & Johnson , H. J. ( 2013 ). Robust multi-site MR data processing: Iterative optimization of bias correction, tissue classification, and registration . Front Neuroinform , 7 , 29 . 10.3389/fninf.2013.00029 24302911 PMC3831347

[b47] Zeun , P. , Lowe , J. , Osborne-Crowley , K. , O’Callaghan , C. , Johnson , E. , Gregory , S. , Nair , A. , Fayer , K. , Rodrigues , F. , Estevez-Fraga , C. , Wild , E. , Zhang , G. , Sampaio , C. , Robbins , T. , Rees , G. , Scahill , R. , Sahakian , B. , & Tabrizi , S. J. ; on Behalf of the HD-YAS Investigators . ( 2018 ). F59 Huntington’s disease young adult study (HD-YAS) . J Neurol Neurosurg Psychiatry , 89 ( Suppl 1 ), A60 – A61 . 10.1136/jnnp-2018-EHDN.160

